# Antimicrobial resistance in ESKAPEE pathogens isolated from institutionalized vs. non-institutionalized elderly patients with urinary tract infections: a 5-years study

**DOI:** 10.1007/s00345-026-06333-0

**Published:** 2026-03-13

**Authors:** Guillermo Ramírez-Vilariño, Iker Alonso-González, Amanda Lopez-Picado, Natalia Burgos-Alonso

**Affiliations:** 1 Department of Public Health, Faculty of Pharmacy, Universidad del País Vasco - Euskal Herriko Unibertsitatea (UPV/EHU), 01006 Vitoria-Gasteiz, Basque Country Spain; 2 Department of Medical Microbiology, Hospital Universitario de Cruces, 48903 Barakaldo, Basque Country Spain; 3 Department of Public Health, Faculty of Medicine, Universidad del País Vasco - Euskal Herriko Uni-bertsitatea (UPV/EHU), 48940 Leioa, Basque Country Spain; 4 Basque Sustainable Pharmacy & Biotherapy Research Group, School of Pharmacy, University of the Basque Country (UPV/EHU), Vitoria-Gasteiz, Spain; 5 Enfermedades del sistema nervioso. Grupo de investigación integradora en salud mental del IIS Biobizkaia. Cruces Plaza, 48903 San Vicente de Barakaldo, Bizkaia Spain

**Keywords:** AMR, Elderly, Epidemiology, ESKAPEE, Institutionalized, UTIs

## Abstract

**Background:**

Urinary tract infections are frequently diagnosed in institutionalized patients, often resulting in unnecessary antibiotic use, especially in asymptomatic cases. This, along with prevalent asymptomatic bacteriuria in the elderly, contributes to antimicrobial resistance, with nursing homes serving as key reservoirs.

**Aim method:**

This study aims to delineate the burden of AMR in ESKAPEE uro-pathogens among elderly patients (aged ≥ 65 years) and to assess the impact of institutionalization on AMR rates, in accordance with EUCAST guidelines.

**Method:**

A retrospective observational study was carried out between 2016 and 2020 using urine culture data obtained from the Microbiology Department of Hospital San Pedro (Spain). Positive urine cultures were reviewed, and isolates belonging to the ESKAPEE group of pathogens were identified and analyzed anonymously.

**Results:**

A total of 34,791 urine cultures were analyzed, of which 26,127 (75.1%) were from the ESKAPEE group. *Escherichia coli* was identified as the most frequently isolated ESKAPEE pathogen (80.5%). Institutionalized patients showed significantly higher AMR rates for numerous antibiotics compared to their non-institutionalized counterparts, particularly manifest in the pronounced resistance rates to fluoroquinolones and a greater prevalence of methicillin-resistant *Staphylococcus aureus *(MRSA) in nursing homes (p<0.001). Additionally, NH settings demonstrated increased rates of multidrug-resistant (MDR) strains of *Escherichia coli* and *Klebsiella pneumoniae*, underscoring a critical challenge in managing UTIs in these environments.

**Conclusion:**

In anticipation of a potential post-antibiotic era, it is imperative to enhance infection control measures and antibiotic stewardship in nursing homes to address the growing burden of MDR bacteria and safeguard patient health.

## Background

Urinary tract infections (UTIs) are among the most common infections in clinical practice, affecting approximately 50% of female patients at least once in their lifetime [1], compared to around 20% in males [2]. The incidence of UTIs increases in elderly patients compared to middle-aged individuals, particularly in those with comorbidities or who are institutionalized [1, 3].

According to Gálvez San Román et al., UTIs account for 3.2% of hospital emergency visits and 22.1% of total infections in these care units, ranking just behind respiratory infections, with a higher prevalence than in the last decade [4]. Gálvez San Román also highlighted the significant role played by the institutionalized patients in the selection of antimicrobial resistance (AMR). In addition, Smithson et al. note that UTI are frequently over diagnosed due to the high prevalence of asymptomatic bacteriuria, leading to unnecessary antimicrobial treatment and the selection of drug-resistant bacterial strains [5].

AMR is defined as the ability of microorganisms to survive or grow despite the presence of drugs designed to inhibit or kill them. AMR represents a serious threat to global health, contributing to increased morbidity and mortality, and is recognized by numerous scientific societies and institutions [6–9]. The Review on Antimicrobial Resistance, published in 2016, estimated that up to 10 million people could die annually from AMR by 2050 [10].

In Europe, according to data published by the European Surveillance of Antimicrobial Consumption Network (ESAC-Net), over 800,000 infections with resistant pathogens oc-curred in 2020 [11], with an estimated death toll exceeding 35,000 [12].

The global impact of AMR is highlighted in the most comprehensive study to date, published in The Lancet [13]. The estimates reported in this study equate the mortality caused by AMR to that of diseases such as HIV or malaria, and potentially even higher, particularly in the developing world. According to the results, of all the possible causes, just six pathogens were responsible for 73.4% of AMR-attributable deaths, in the following order: *Escherichia coli*,* Staphylococcus aureus*,* Klebsiella pneumoniae*,* Streptococcus pneumoniae*,* Acinetobacter baumanii* and *Pseudomonas aeruginosa*, most of which are included within the ESKAPE group.

In 2008, Rice defined the ESKAPE group as a coterie of bacteria with notable virulence and the ability to develop AMR [14]. The acronym ESKAPE stands for *Enterococcus faecium*,* Staphylococcus aureus*,* Klebsiella pneumoniae*,* Acinetobacter baumanii*,* Pseudomonas aeruginosa* and *Enterobacter* species. *E. coli* is another common human pathogen that tends to develop AMR, particularly in UTIs; hence, some authors include it in an expanded acronym, ESKAPEE [15–17].

Recently, in 2024, the WHO updated the Bacteria Priority Pathogen List, which was first published in 2017 [18]. The aim of this list is to guide source allocation, promote and direct R&D of novel antibacterial agents and support the development of effective strategies to prevent, control, and treat infections caused by priority pathogens. The list includes the ESKAPEE pathogens, among others.

Understanding the local epidemiology is essential for adapting and optimizing antimicrobial treatment [19]. Furthermore, focusing efforts on the ESKAPEE bacteria is crucial for enhancing treatment efficacy, achieving better results, and optimizing the use of time and resources, which is particularly important in infections treated empirically, such as UTIs. Nevertheless, the local epidemiological available data on elderly patients are short and they do not differentiate between institutionalized and non-institutionalized patients.

Elderly patients are considered a high-risk group for developing healthcare-associated infections due to age-related immune dysfunction (immunosenescence) [20]. Comorbidities can further complicate infections and reduce the effectiveness of treatments. UTIs are frequently diagnosed among residents of long-term care facilities; of-ten leading to the routine prescription of antibiotics, even in asymptomatic cases [21]. The increasing prevalence of asymptomatic bacteriuria in the elderly further complicates diagnosis, frequently resulting in the over diagnosis of UTIs and the subsequent unnecessary administration of treatment [22]. Such unnecessary treatment, or even inadequate empirical antibiotic therapy, can contribute to the selection of resistant strains, thereby exacerbating the issue of AMR [23]. Nursing Homes (NH) are considered a major reservoir of AMR [24, 25]. According to Cassone and Mody, over 35% of NHs residents are colonized with multidrug-resistant (MDR) organisms. Gajdács et al. reported significant differences in the resistance rates among inpatients and outpatients isolates for several antibiotics, with higher rates of resistance to nitrofurantoin, fosfomycin and trime-thoprim-sulfamethoxazole than previously reported in the same geographical region [26].

The purpose of this 5-years study was to report the AMR profiles of the ESKAPEE bacteria isolated from urine cultures of elderly patients in La Rioja, Spain, be-tween 2016 and 2020, both inclusive, and to determine if there were significant differences in the AMR patterns between institutionalized and non-institutionalized elderly patients, as expected.

## Methods

### Study design

This retrospective observational study was conducted from 2016 to 2020, both inclusive, at hospital San Pedro in Logroño, La Rioja, Spain. Approved by the Ethics Committee (Drug Research Ethics Committee of La Rioja (CEImLAR) (protocol code EPA-OD-111)), the study aimed to describe the burden of AMR in ESKAPEE uropathogens among elderly patients in La Rioja, Spain, and to evaluate the impact of institutionalization on AMR rates. According to the Ethics Committee’s ruling, informed consent was not necessary since the data was collected anonymously.

According to the National Statistics Institute (INE) [52], in January 2022, la Rioja re-ported 319,892 inhabitants (0.67% of the population of Spain), of which 68,851 were aged 65 years or older (21.5%). Of these, 38,144 were female (55.4%). In contrast, in January 2012, la Rioja reported 323,609 inhabitants (0.68% of the population of Spain), of which 60,270 were elderly people (18.6%). This represents a 15.6% increase in the elderly population over ten years. The most recent report on institutionalized patients in La Rioja indicated that there were 2,503 individuals in 2011 [53]. The same report showed a ratio of 2.18 female patients for every male.

### Urine cultures

Urine culture data were obtained from the Microbiology Department at Hospital San Pedro in Logroño, Spain, between 2016 and 2020, both inclusive. Patients aged 65 years or older were included. Among all these positive urine cultures, ESKAPEE isolated pathogens were selected and analyzed anonymously. A positive culture was considered with a cutoff at > 10^5 CFU/ml.

Raw data were collected, including the isolation date, patient gender and age, medical unit, institutionalization status, isolated pathogen, and minimum inhibitory concentration (MIC) of the tested antimicrobials. Institutionalized patients are usually dependent people who live day and night in NH. Duplicate cultures (same pathogen and antibiotype) were excluded by selecting only the first isolate within a 3-month period; cultures collected after this period were considered as new infections.

To simplify statistical analysis and comparisons, the MIC values were recoded to Susceptible at standard dosing regimen (S), susceptible at Increase exposure (I), and Resistant (R), according to the clinical breakpoints published by the EUCAST, version 14.0, valid from January 1, 2024 [54]. The most recent breakpoints were selected to ensure clinically relevant responses to this longstanding issue, prioritizing MIC values over simplistic S/I/R categorizations. Fosfomycin breakpoints underwent major updates (values < 64 µg/L previously deemed susceptible without finer gradient distinctions), so pre-update criteria were applied to maintain a viable E. coli sample size for analysis. Retesting with precise MICs could reveal higher resistance rates by reclassifying borderline cases.

For the definitions of MDR, extensively drug-resistant (XDR) and pandrug-resistant (PDR) bacteria, the criteria proposed by Magiorakos et al. [55] were followed.

### Statistical analysis

To perform statistical analyses, SPSS software version 23 (IBM Corp., Endicott, NY, USA) was used. The χ2 test and Mann-Whitney U test were employed to assess the significance of associations. Multivariable logistic regression models were constructed with simultaneous entry of residence, sex, and age (3 categories) to assess independent predictors of resistance to ciprofloxacin, fosfomycin, and nitrofurantoin. Odds ratios (OR), 95% confidence intervals, Wald statistics, and p-values were calculated for all variables, with statistical significance defined as *p* < 0.001 across models.

## Results

### Baseline characteristics

During the study period, 34,791 positive urine cultures were evaluated according to their microbiological standards. Among these samples, 26,127 (75.1%) ESKAPEE pathogens were isolated. The ESKAPEE pathogens isolated from the urine cultures are shown in Table 1. *Escherichia coli* was the most frequently detected ESKAPEE uropathogen (80%), followed by *Klebsiella pneumoniae* (10%) and *Pseudomonas aeruginosa* (4%). In the institutionalized patient group (*n* = 3,551), the median age was 88 years (IQR: 84–92), while in the non-institutionalized patient group (*n* = 22,576), the median age was 80 years (73–86). Females presented UTIs with a higher frequency in both cases: 2,906 (81.8%) in institutionalized patients and 16,983 (75.2%) in non-institutionalized patients. AMR of microorganisms is listed in Table 2.

**Table 1:** Distribution of the ESKAPEE pathogens among the urinary isolates

Microorganisms	Institutionalized patient (*n*, %)	Non-institutionalized patient (*n*, %)	Overall (*n*, %)
*Escherichia coli*	3,008 (84.7%)	18,017 (79.8%)	21,025 (80.5%)
*Klebsiella pneumoniae*	306 (8.6%)	2,369 (10.5%)	2,675 (10.2%)
*Pseudomonas aeruginosa*	110 (3.1%)	866 (3.8%)	976 (3.7%)
*Enterobacter spp.*	33 (0.9%)	712 (3.2%)	745 (2.9%)
*Staphylococcus aureus*	44 (1.2%)	317 (1.4%)	361 (1.4%)
*Enterococcus faecium*	49 (1.4%)	282 (1.2%)	331 (1.3%)
*Acinetobacter baumanii*	1 (0.0%)	13 (0.1%)	14 (0.1%)
Overall	3,551	22,576	26,127

**Table 2:** Antimicrobial resistance of microorganisms (in percentage)

Microorganisms Antimicrobials	E. faecium (*n* = 331)	S. aureus (*n* = 361)	K. pneumoniae (*n* = 2,675)	A. baumanii (*n* = 14)	*P*. aeruginosa (*n* = 976)	Enterobacter spp. (*n* = 745)	E. coli (*n* = 21,025)
PEN	-	82.8%	-	-	-	-	-
OXA	-	39.6%	-	-	-	-	-
AMC	82.9%	-	12.8%	-	-	91.0%	26.0%
TZP		-	9.2%	-	13.7%	21.9%	10.5%
FOX	-	41.1%	8.3%	-	-	91.1%	5.9%
CFX	-	-	13.5%	-	-	73.4%	23.5%
CTX	-	-	8.7%	-	-	32.2%	19.8%
CAZ	-	-	8.1%	-	13.2%	27.4%	15.0%
CTL	-	0.6%	-	-	-	-	-
ETP	-	-	1.6%	-	-	4.1%	0.0%
IPM	-	-	0.8%	21.4%	10.9%	0.8%	0.0%
MEM	-	-	0.7%	21.4%	5.9%	0.8%	0.0%
CIP	90.6%	53.4%	17.3%	14.3%	43.5%	14.0%	46.0%
LVX	87.3%	53.4%	14.4%	15.4%	38.7%	10.5%	44.7%
NOR	-	-	26.5%	-	-	21.0%	52.0%
AMK	-	-	0.8%	21.4%	6.1%	2.5%	6.0%
GEN	-	16.8%	5.3%	23.1%	21.6%	5.6%	19.8%
TOB	-	32.3%	8.1%	21.4%	25.1%	6.0%	25.6%
VAN	0 0.3%	0.3%	-	-	-	-	-
TEI	0.9%	1.1%	-	-	-	-	-
LZD	0.3%	0.3%	-	-	-	-	-
DAP	-	1.7%	-	-	-	-	-
ERY	-	23.2%	-	-	-	-	-
CLI	-	27.8%	-	-	-	-	-
TET	-	3.8%	-	-	-	-	-
SXT	-	0.6%	11.5%	50.0%	-	7.5%	33.6%
FOS	-	-	37.2%	-	-	39.1%	9.7%
NIT	-	82.8%	53.5%	-	-	69.3%	5.1%

*PEN*: penicillin, *OXA*: oxacillin, *AMC*: amoxicillin-clavulanate, *TZP*: piperacil-lin-tazobactam, *FOX*: cefoxitin, *CFX*: cefuroxime, *CTX*: cefotaxime, *CAZ*: ceftazidime, *CTL*: ceftolozane-tazobactam, *ETP*: ertapenem, *IPM*: imipenem, *MEM*: meropenem, *CIP*: ciprofloxacin, *LVX*: levofloxacin, *NOR*: norfloxacin, *AMK*: amikacin, *GEN*: gentamicin, *TOB*: tobramycin, *VAN*: vancomycin, *TEI*: teicoplanin, *LZD*: linezolid, *DAP*: daptomycin, *ERY*: erythromycin, *CLI*: clindamycin, *TET*: tetracycline, *SXT*: trimethoprim-sulfamethoxazole, *FOS*: fosfomycin, *NIT*: nitrofurantoin

### *Enterococcus faecium*

49 and 282 isolates corresponded to Enterococcus faecium in the institutionalized and non-institutionalized patient group, respectively. Differences in AMR in both groups are shown in Table 3. In institutionalized patients, all isolates (48/48, with 1 missing value) were resistant to ciprofloxacin and levofloxacin, whereas in non-institutionalized patients, the resistance rate were 88.9% and 85.0% (*p* < 0.05 and *p* < 0.01), respectively. No other relevant differences were observed. Remarkably, the resistance rate to vancomycin and linezolid were very low, with only one isolate of all samples being resistant to both antibiotic.

**Table 3:** Antimicrobial resistance rates in *Enterococcus faecium*

E. faecium	Institutionalized patients		Non-institutionalized patients		*p* Value
	*n* = 49	%	*n* = 282	%	
AMC	39/45	86.7%	222/270	82.2%	0.386
CIP	48/48	100%	241/271	88.9%	0.016
LVX	48/48	100%	233/274	85.0%	0.004
VAN	0/48	0.0%	1/276	0.4%	0.677
TEI	0/48	0.0%	3/271	1.1%	0.465
LZD	0/48	0.0%	1/275	0.4%	0.676

*AMC*: amoxicillin-clavulanate, *CIP*: ciprofloxacin, *LVX*: levofloxacin, *VAN*: van-comycin, *TEI*: teicoplanin, *LZD*: linezolid

### *Staphylococcus aureus*

A total of 44 isolates corresponded to *S. aureus* in the institutionalized patient group, while 317 were from the non-institutionalized patient group. The prevalence of methicillin-resistant S. aureus (MRSA), inferred from the results of cefoxitin resistance rates, in institutionalized patients was significantly higher than in non-institutionalized patients: 65.9% vs. 37.8% (*p* = 0.001), respectively. Fluoroquinolones (ciprofloxacin and levofloxacin) also demonstrated significantly higher resistance rates in institutionalized patients: 73.8% and 74.4% vs. 50.5% and 50.5% (*p* = 0.005 and *p* = 0.03), respectively. *S. aureus* showed similarly high resistance rates to penicillin in both groups (*p* > 0.01). The detail AMR rates are presented in Table 4.

**Table 4:** Antimicrobial resistance rates in *Staphylococcus aureus*

S. aureus	Institutionalized patients		Non-institutionalized patients		*p* Value
	*n* = 44	%	*n* = 317	%	
PEN	37/41	90.2%	251/307	81.8%	0.177
OXA	27/43	62.8%	112/308	36.4%	0.001
FOX	27/41	65.9%	116/307	37.8%	0.001
CTL	0/41	0.0%	2/297	0.7%	0.600
CIP	31/42	73.8%	152/301	50.5%	0.005
LVX	32/43	74.4%	156/309	50.5%	0.003
GEN	6/42	14.3%	53/309	17.2%	0.642
TOB	16/41	39.8%	97/309	31.4%	0.327
VAN	0/42	0.0%	1/308	0.3%	0.712
TEI	0/40	0.0%	4/308	1.3%	0.469
LZD	0/42	0.0%	1/308	0.3%	0.712
DAP	1/42	2.4%	5/308	1.6%	0.723
ERY	13/41	31.7%	38/308	22.1%	0.171
CLI	14/41	34.1%	83/308	26.9%	0.334
TET	2/41	4.9%	11/300	3.7%	0.704
SXT	0/43	0.0%	2/309	0.6%	0.517

*PEN*: penicillin, *OXA*: oxacillin, *FOX*: cefoxitin, *CTL*: ceftolozane-tazobactam, *CIP*: ciprofloxacin, *LVX*: levofloxacin, *GEN*: gentamicin, *TOB*: tobramycin, *VAN*: vancomycin, *TEI*: teicoplanin, *LZD*: linezolid, *DAP*: daptomycin, *ERY*: erythromycin, *CLI*: clindamycin, *TET*: tetracycline, *SXT*: trimethoprim-sulfamethoxazole

### *Klebsiella pneumoniae*

After *E. coli*,* K. pneumoniae* was the second most frequently isolated uropathogen: 306 isolates in institutionalized patients and 2369 in non-institutionalized patients. Except for nitrofurantoin (*p* > 0.05), *K. pneumoniae* showed higher resistance rates for all others antibiotics tested in the institutionalized patients (amikacin *p* < 0.05, gentamicin *p* = 0.001, rest *p* < 0.001). Table 5 shows the AMR rates in *K. pneumoniae*.

**Table 5:** Antimicrobial resistance rates in Klebsiella pneumoniae

K. pneumoniae	Institutionalized patients		Non-institutionalized patients		*p* Value
	*n* = 306	%	*n* = 2,369	%	
AMC	76/287	26.5%	263/2,355	11.2%	< 0.001
TZP	46/288	16.0%	197/2,357	8.4%	< 0.001
FOX	49/288	17.0%	169/2,353	7.2%	< 0.001
CFX	78/287	27.2%	279/2,353	11.9%	< 0.001
CTX	64/287	22.3%	167/2,357	7.1%	< 0.001
CAZ	60/288	20.8%	153/2,357	6.5%	< 0.001
ETP	18/288	6.3%	24/2,356	1.0%	< 0.001
IPM	9/288	3.1%	13/2,356	0.6%	< 0.001
MEM	6/284	2.1%	11/2,320	0.5%	< 0.001
CIP	106/287	36.9%	352/2,357	14.9%	< 0.001
LVX	90/284	31.7%	286/2,320	12.3%	< 0.001
NOR	133/283	47.0%	560/2,335	24.0%	< 0.001
AMK	5/284	1.8%	15/2,319	0.6%	0.043
GEN	27/288	9.4%	114/2,356	4.8%	0.001
TOB	50/288	17.4%	163/2,357	6.9%	< 0.001
SXT	71/287	24.7%	232/2,358	9.8%	< 0.001
FOS	167/287	58.2%	816/2356	34.6%	< 0.001
NIT	148/287	51.6%	1,264/2,354	53.7%	0.495

*AMC*: amoxicillin-clavulanate, *TZP*: piperacillin-tazobactam, *FOX*: cefoxitin, *CFX*: cefuroxime, *CTX*: cefotaxime, *CAZ*: ceftazidime, *ETP*: ertapenem, *IPM*: imipenem, *MEM*: meropenem, *CIP*: ciprofloxacin, *LVX*: levofloxacin, *NOR*: norfloxacin, *AMK*: amikacin, *GEN*: gentamicin, *TOB*: tobramycin, *SXT*: trimethoprim-sulfamethoxazole, *FOS*: fosfomycin, *NIT*: ni-trofurantoin

### *Acinetobacter baumanii*

As shown in Table 1, only one *A. baumanii* was isolated from urine cultures of institutionalized patients. Comparisons were not relevant.

### *Pseudomonas aeruginosa*

A total of 976 *P. aeruginosa* isolates were observed, with 110 and 866 isolates corresponding to institutionalized and non-institutionalized patients, respectively. No significant differences were reported between the AMR rates in Pseudomonas aeruginosa urine cultures. Results are shown in Table 6.

**Table 6:** Antimicrobial resistance rates in Pseudomonas aeruginosa

*P*. aeruginosa	Institutionalized patients		Non-institutionalized patients		*p* Value
	*n* = 110	%	*n* = 866	%	
TZP	14/106	13.2%	118/858	13.8%	0.878
CAZ	14/106	13.2%	113/858	13.2%	0.991
IPM	9/106	8.5%	96/860	11.2%	0.405
MEM	5/106	4.7%	52/854	6.1%	0.874
CIP	54/106	50.9%	365/858	42.5%	0.1
LVX	49/106	46.2%	321/850	37.8%	0.092
AMK	8/106	7.5%	50/849	5.9%	0.501
GEN	22/102	21.6%	185/855	21.6%	0.987
TOB	29/106	27.4%	213/857	24.9%	0.575

*TZP*: piperacillin-tazobactam, *CAZ*: ceftazidime, *IPM*: imipenem, *MEM*: mero-penem, *CIP*: ciprofloxacin, *LVX*: levofloxacin, *AMK*: amikacin, *GEN*: gentamicin, *TOB*: tobramy-cin

### *Enterobacter spp*

*Enterobacter* species accounted for 33 and 712 isolates from institutionalized and non-institutionalized patients, respectively. Fluoroquinolones resistance rates were higher in institutionalized patients compared to non-institutionalized patients: ciprofloxacin resistance rates were 35.5% vs. 13.0% (*p* < 0.001), levofloxacin 32.3% vs. 9.5% (*p* < 0.001), and norfloxacin 43.3% vs. 20.0% (*p* < 0.005). The resistance rates to third-generation cephalosporins were significantly higher in institutionalized patients compared to non-institutionalized patients: cefotaxime resistance rates were 56.7% vs. 31.2% (*p* = 0.005), ceftazidime resistance rates 45.2% vs. 26.6% (*p* < 0.05), respectively. In addition, fosfomycin showed higher resistance rates in institutionalized patients: 58.1% vs. 38.2% (*p* < 0.05), respectively (table 7).

**Table 7:** Antimicrobial resistance rates in *Enterobacter spp*

Enterobacter spp.	Institutionalized patients		Non-institutionalized patients		*p* Value
	*n* = 33	%	*n* = 712	%	
AMC	29/31	93.5%	642/706	90.9%	0.618
TZP	7/31	22.6%	154/705	21.8%	0.923
FOX	30/31	96.8%	636/700	90.9%	0.258
CFX	26/31	83.9%	512/702	72.9%	0.178
CTX	17/30	56.7%	220/706	31.2%	0.005
CAZ	14/31	45.2%	188/706	26.6%	0.024
ETP	1/31	3.2%	29/704	4.1%	0.806
IPM	0/31	0.0%	6/701	0.9%	0.395
MEM	0/31	0.0%	6/695	0.9%	0.604
CIP	11/31	35.5%	92/706	13.0%	< 0.001
LVX	10/31	32.3%	66/695	9.5%	< 0.001
NOR	13/30	43.3%	139/694	20.0%	0.002
AMK	1/31	3.2%	17/696	2.4%	0.784
GEN	2/31	6.5%	39/706	5.5%	0.826
TOB	2/31	6.5%	42/706	5.9%	0.908
SXT	4/31	12.9%	51/706	7.2%	0.111
FOS	18/31	58.1%	270/706	38.2%	0.027
NIT	22/31	71.0%	484/706	69.3%	0.840

*AMC*: amoxicillin-clavulanate, *TZP*: piperacillin-tazobactam, *FOX*: cefoxitin, *CFX*: cefuroxime, *CTX*: cefotaxime, *CAZ*: ceftazidime, *ETP*: ertapenem, *IPM*: imipenem, *MEM*: meropenem, *CIP*: ciprofloxacin, *LVX*: levofloxacin, *NOR*: norfloxacin, *AMK*: amikacin, *GEN*: gentamicin, *TOB*: tobramycin, *SXT*: trimethoprim-sulfamethoxazole, *FOS*: fosfomycin, *NIT*: ni-trofurantoin

### *Escherichia coli*

Escherichia coli was the most prevalent pathogen isolated from urine cultures ac-counting for a total of 21,025, representing 60.4% of all urine samples during in the study period (80.5% of ESKAPEE uropathogens). Institutionalized patients showed higher resistance rates for all tested antibiotics compare to non-institutionalized patients (*p* < 0.001), excepting for carbapenems, for which no resistance was observed in urine samples. Resistance rates of beta-lactam antibiotics were twice as high in the institutionalized patients (three times higher for third-generation cephalosporins): amoxicillin-clavulanate 45.3% vs. 23.0%, piperacillin-tazobactam 17.2% vs. 9.5%, cefoxitin 10.4% vs. 5.2%, cefuroxime 58.3% vs. 18.1%, cefotaxime 54.0% vs. 14.4%, ceftazidime 44.7% vs. 10.3%. Fluoroquinolones showed significantly higher resistance rates in institutionalized patients: ciprofloxacin 76.9% vs. 41.1%, levofloxacin 74.8% vs. 39.9%, norfloxacin 81.0% vs. 47.5%. AMR rates for aminoglycoside antibiotics increased in institutionalized patients: amikacin 12.8% vs. 4.9%, gentamicin 35.9% vs. 17.3%, tobramycin 48.5% vs. 22.0%. Other antimicrobials showed higher resistance rates in institutionalized patients: sulfamethoxa-zole-trimethoprim 50.1% vs. 31.0%, fosfomycin 20.1% vs. 8.0%, nitrofurantoin 10.8% vs. 4.2%. The results are summarized in Table 8.

**Table 8:** Antimicrobial resistance rates in *Escherichia coli*

E. coli	Institutionalized patients		Non-institutionalized patients		*p* Value
	*n* = 3,008	%	*n* = 18,017	%	
AMC	1,276/2,815	45.3%	4,114/17,897	23.0%	< 0.001
TZP	483/2,814	17.2%	1,693/17,888	9.5%	< 0.001
FOX	292/2,812	10.4%	929/17,880	5.2%	< 0.001
CFX	1,639/2,809	58.3%	3,227/17,870	18.1%	< 0.001
CTX	1,521/2,816	54.0%	2,570/17,895	14.4%	< 0.001
CAZ	1,258/2,816	44.7%	1,843/17,893	10.3%	< 0.001
ETP	1/2,818	0.0%	7/17,895	0.0%	0.927
IPM	1/2,816	0.0%	0/17,895	0.0%	0.828
MEM	1/2,786	0.0%	0/17,680	0.0%	0.133
CIP	2,166/2,816	76.9%	7,360/17,897	41.1%	< 0.001
LVX	2,084/2,785	74.8%	7,057/17,679	39.9%	< 0.001
NOR	2,280/2,815	81.0%	8,486/17,870	47.5%	< 0.001
AMK	357/2,785	12.8%	861/17,680	4.9%	< 0.001
GEN	1,012/2,817	35.9%	3,089/17,894	17.3%	< 0.001
TOB	1,367/2,816	48.5%	3,935/17,895	22.0%	< 0.001
SXT	1,412/2,817	50.1%	5,548/17,897	31.0%	< 0.001
FOS	565/2,817	20.1%	1,438/17,898	8.0%	< 0.001
NIT	303/2,817	10.8%	759/17,898	4.2%	< 0.001

*AMC*: amoxicillin-clavulanate, *TZP*: piperacillin-tazobactam, *FOX*: cefoxitin, *CFX*: cefuroxime, *CTX*: cefotaxime, *CAZ*: ceftazidime, *ETP*: ertapenem, *IPM*: imipenem, *MEM*: meropenem, *CIP*: ciprofloxacin, *LVX*: levofloxacin, *NOR*: norfloxacin, *AMK*: amikacin, *GEN*: gentamicin, *TOB*: tobramycin, *SXT*: trimethoprim-sulfamethoxazole, *FOS*: fosfomycin, *NIT*: ni-trofurantoin

### Multidrug resistant bacteria

As shown in Table 4, the prevalence of MDR bacteria varied between institutionalized and non-institutionalized patients: *Enterobacter* species and *Pseudomonas aeruginosa* did not show significant differences between the groups, whereas *Klebsiella pneumoniae* and Escherichia coli had higher rates of MDR in institutionalized patients, at 30.1% vs. 13.5% (*p* < 0.001) and 60.7% vs. 26.1% (*p* < 0.001), respectively.

### Multivariant analysis

A multivariate analysis was conducted on the most relevant antibiotic-pathogen pairs in our setting: E. coli-ciprofloxacin, E. coli-fosfomycin, and E. coli-nitrofurantoin, including confounding variables age, sex, and whether the patient resides in a nursing home (dependent variable).

Multivariable logistic regression models identified residence as the strongest independent predictor of resistance to ciprofloxacin (OR 4.51, 95% CI 4.10–4.97), fosfomycin (OR 2.50, 95% CI 2.24–2.80), and nitrofurantoin (OR 2.56, 95% CI 2.21–2.97), remaining significant after adjusting for age and sex (all *p* < 0.001). Female sex was protective for ciprofloxacin (OR 0.47, 95% CI 0.44–0.51) and nitrofurantoin (OR 0.57, 95% CI 0.50–0.66) but absent from the fosfomycin model. Age (3 categories) consistently increased resistance risk across antibiotics (OR 1.17–1.25, all *p* < 0.001) Table 9.

**Table 9:** Rates of MDR in *Enterobacteriaceae* and *Pseudomonas aeruginosa*

Microorganism	Institutionalized patients		Non-institutionalized patients		*p* Value
	*n*	%	*n*	%	
*Klebsiella pneumoniae*	92/306	30.1%	320/2,369	13.5%	< 0.001
*Enterobacter spp*.	27/33	81.8%	550/712	77.2%	0.539
*Escherichia coli*	1,825/3,008	60.7%	4,702/18,017	26.1%	< 0.001
*Pseudomonas aeruginosa*	12/110	10.1%	113/866	13.0%	0.527

## Discussion

UTIs are a common condition seen in primary care and their incidence is directly related to aging [27]. In elderly patients, symptoms of UTI may present atypically and include hypotension, tachycardia, urinary incontinence, poor appetite, drowsiness, frequent falls, and delirium [28]. Furthermore, asymptomatic bacteriuria is observed in 20% of older women living in the community and 50% of those in NH [29]. Additionally, limited access to timely laboratory testing often leads to prolonged empirical antibiotic therapy [24]. The overuse and misuse of antibiotic treatments have further exacerbated the problem of AMR [22, 30]. The increasing prevalence of AMR has created an urgent need for programs and interventions designed to optimize antimicrobial use, commonly referred to as anti-microbial stewardship programs [31–33].

In this context, we developed this study to determine the burden of AMR in NH and to compare it with AMR rates among non-institutionalized patients, with results corroborating our expectations. During a 5-years period (2016–2020), 34,791 urine cultures obtained from elderly patients in La Rioja, Spain, were characterized and analyzed according to European Committee on Antimicrobial Susceptibility Testing (EUCAST) guidelines [11]. Of these, 26,127 corresponded to ESKAPEE bacteria, E. coli being the most frequently isolate, in accordance with other published case series [34–36].

Significant differences were observed in the AMR rates between institutionalized and non-institutionalized patients, confirming our initial hypothesis. Notably, AMR rates for fluoroquinolones (ciprofloxacin, levofloxacin, norfloxacin) were concerning: E. coli and K. pneumoniae exhibited nearly twice the resistance rate in NH compared to non-institutionalized patients (ciprofloxacin resistance rates: 76.9% vs. 41.1% for E. coli and 36.9% vs. 14.9% for K. pneumoniae). According to ECDC reported data, the fluoroquinolone resistance rates in the elderly in 2020 in Spain were 30.1% and 27.1%, respectively [37]. Additionally, Laffont-Lozes et al. reported that decreasing AMR rates to fluoroquinolones were correlated with reduced use of these antibiotics [38].

Multivariable data reveal significantly elevated odds of resistance to ciprofloxacin (OR 4.51), fosfomycin (OR 2.50), and nitrofurantoin (OR 2.56) in patients residing in nursing homes compared to the community, persisting after adjustment for age and sex (all *p* < 0.001). This independent association underscores specific risk factors in institutional settings, such as higher antibiotic use, nosocomial colonization, or shared hygiene practices.

Regarding sex, greater resistance is observed in men for ciprofloxacin (OR 0.47 in women) and nitrofurantoin (OR 0.57 in women), with no significant effect for fosfomycin. This difference may be explained by prescribing patterns: fosfomycin is typically given as a single dose (or two), without sex differences, whereas ciprofloxacin in men requires prolonged treatments (2–4 weeks) to cover prostatitis versus 3–5 days for uncomplicated UTIs in women, increasing selective pressure for resistance.

The prevalence of MRSA in NH is notable at 65.9%, which is consistent with other case series reported [39]. Colonized individuals, including healthcare personnel and visitors, contribute to the dissemination of MRSA within these facilities [40, 41]. Routine screenings could be recommended to identify carriers.

In *Enterobacteriaceae*, the production of extended-spectrum β-lactamase (ESBL) is an alarmingly growing issue [42–44]. Differences observed in AMR rates for many antibiotics —beyond penicillins and cephalosporines, including fluoroquinolones, aminoglycosides, sulfamethoxazole-trimethoprim [42]— can be explained by ESBL-producing strains.On the other hand, inducible chromosomal AmpC-producing Enterobacter species have been described [45]. The overuse of broad-spectrum cephalosporins or penicillin-inhibitor combinations, often in NH, may result in the expression of AmpC, which can account for the higher resistant rate to cefotaxime [46]. An identical AMR-producing mechanism has been shown in Pseudomonas [47].

Fosfomycin is the most frequently prescribed antibiotic for the treatment of UTIs due to its effectiveness and the rarity of resistance in E. coli [48]. However, its overuse has led to the development of AMR [49]. Indeed, Loras et al. have raised concerns about the potential dissemination of plasmid-mediated fosfomycin resistance by fosA3 [50]. Consequently, according to Huttner et al. [51], for institutionalized females with acute simple cystitis and no risk factors for a MDR gram-negative infection, we suggest nitrofurantoin. Fosfomycin should be reserved to targeted antibiotic therapies due to its increasing AMR rates.

Based on the results presented, increased attention should be given to the diagnosis and management of UTIs in elderly patients, particularly within NH settings. AMR is becoming a significant concern in these environments, and it is the responsibility of all healthcare professionals to mitigate the emergence of AMR and to preserve the useful lifespan of antibiotics.

Some limitations must be acknowledged. Susceptibility tests were not performed for all isolates or all antimicrobials. Consequently, some isolates had missing values for AMR rates: records with more than 10% missing values were excluded from the analysis. The results were expressed as a percentage of the valid values. Furthermore, the production of ESBLs or carbapenemases could not be determined from the extracted data.

Additionally, EUCAST updated the MIC breakpoints for fosfomycin in E. coli in 2021. Since the study period covered 2016–2020, the urine samples were reported according to the previous standards, making it impossible to assess AMR rates using the current valid criteria. Therefore, 2020 fosfomycin MICs breakpoint were applied for E. coli isolates (breakpoint tables for interpretation of MICs and zone diameters, version 10.0) [56]. Moreover, as EUCAST guidelines only define MIC breakpoints for fosfomycin in E. coli, the fosfomycin MIC breakpoint for Enterobacteriaceae was based on the clinical breakpoint established for E. coli.

Following the recommendations of Magiorakos, it was not possible to determine the presence of XDR and PDR bacteria due to incomplete susceptibility testing.

Apart from that, the interpretation of fosfomycin resistance has specific limitations. Due to the retrospective nature of the data, based on automated systems reporting bounded MIC values (e.g., < 64 mg/L), current methodological standards (EUCAST 2024)—which often require precise MIC values—could not be applied. Therefore, the reported fosfomycin resistance rates reflect historical detection criteria and should be interpreted cautiously in the context of current practice.

## Conclusions

In conclusion, the aim of the study was to investigate the epidemiology of UTIs and AMR rates in NH in La Rioja, Spain, and to compare these findings with those in elderly non-institutionalized patients. Consistent with global patterns, we observed higher AMR rates in institutionalized patients compared to elderly individuals living in the community.

Facing a post-antibiotic era, infection control measures and effective antibiotic stewardship programs are crucial for combating the burden of AMR and mitigating its impact on patient health in institutional settings. Since NH has become bastions of multi-drug-resistant bacteria, it is essential to enhance our efforts in these healthcare settings.

## Data Availability

The raw data supporting the conclusions of this article will be made available by the authors on request.
